# Recovery of Phenolic Compounds with Antioxidant Capacity Through Solid-State Fermentation of Pistachio Green Hull

**DOI:** 10.3390/microorganisms13010035

**Published:** 2024-12-27

**Authors:** Andrés Javier Ordoñez-Cano, Ulises Ramírez-Esparza, Fernando Méndez-González, Mónica Alvarado-González, Ramiro Baeza-Jiménez, Leonardo Sepúlveda-Torre, Lilia Arely Prado-Barragán, José Juan Buenrostro-Figueroa

**Affiliations:** 1 Biotechnology and Bioengineering Laboratory, Centro de Investigación en Alimentación y Desarrollo, Delicias 33089, Chihuahua, Mexico; aordonez224@estudiantes.ciad.mx (A.J.O.-C.); uramirez223@estudiantes.ciad.mx (U.R.-E.); fernando.mendez@ciad.mx (F.M.-G.); ramiro.baeza@ciad.mx (R.B.-J.); 2 Microbiology and Molecular Biology Laboratory, Centro de Investigación en Alimentación y Desarrollo, Delicias 33089, Chihuahua, Mexico; salvarado@ciad.mx; 3 Food Research Department, School of Chemistry, Universidad Autónoma de Coahuila, Saltillo 25280, Coahuila, Mexico; leonardo_sepulveda@uadec.edu.mx; 4 Solid Fermentations Pilot Plant, Biotechnology Department, Universidad Autónoma Metropolitana–Iztapalapa, Av. San Rafael Atlixco 186, Col. Vicentina, Ciudad de México 09340, Mexico

**Keywords:** bioprocesses, gallic acid, geranine, *Aspergillus niger* GH1, pistachio waste

## Abstract

Pistachio green hull (PGH) represents the non-edible fraction obtained after the seed is harvested and is an important source of phenolic compounds. Solid-state fermentation (SSF) is a viable biotechnological and economical technique for extracting phenolic compounds. This study aimed to evaluate the SSF with *Aspergillus niger* GH1 to recover total phenolic compounds (TPC) with antioxidant capacity (AC) from PGH. For this, the time of higher TPC and AC (DPPH [2,2-diphenyl-1-picrylhydrazyl], ABTS [2,2-azinobis-(3-ethylbenzothiazoline-6-sulfonate)], FRAP [ferric reducing antioxidant power]) was selected. Then, moisture, inoculum concentration, and aeration rate were evaluated. *A. niger* GH1 was able to grow and colonize the PGH, with the higher value of TPC (23.83 mg/g of dry mass (gdm)) obtained after 24 h of culture, which significantly correlated with AC (Pearson’s R = 0.69). Moisture and aeration rate were the main factors influencing TPC. The highest values for both TPC and AC were achieved in treatment 8 (60% moisture, 5 × 10^6^ spores/mL, and 1 L/Kgwm min), resulting in a 129% and 1039% increase, respectively. Gallic acid 4-*O*-glucoside and geranine were identified in the PGH extracts using high-performance liquid chromatography coupled with mass spectrometry. The SSF provides eco-friendly alternatives for releasing bioactive compounds from PGH, adding value to this waste.

## 1. Introduction

The pistachio (*Pistacia vera* L.) is one of the most important dried fruits today, and there are some reports related to its consumption since three hundred thousand years ago [1,2]. Its consumption occurs as snacks, oils, or flavoring for cakes, cookies, and bread [3]. Thanks to its wide demand, by 2023 a global production of 1.1 million tons was estimated, where the main producing countries were the United States, Iran, Turkey, and Syria [4].

Consumption of this product is primarily driven by its culinary properties, but nowadays, consumers also consider its numerous health benefits. It possesses anti-diabetic, cardiovascular, antioxidant, anti-inflammatory, anticancer, and anti-obesogenic properties [3]. Furthermore, it contains 18–23% protein, 18% carbohydrates, and a lower percentage of fiber, minerals, and bioactive compounds, such as TPC [1,5,6].

Pistachio consumption produces two by-product fractions: the first is the edible fraction, which is the commercial fruit, and the second is a residue, known as PGH, which makes up 45 to 60% of the total fruit. Unfortunately, this significant portion is currently not utilized, highlighting the issue of food waste and the need for sustainable practices in the food industry [7,8,9,10]. Finding alternative uses for PGH is essential because its high moisture content promotes the growth of contaminating microorganisms, and its high TPC amount leads to soil alterations in the area where it is deposited, causing environmental issues [9].

Several studies have reported that PGH is a potential source of TPC with antioxidant, antimicrobial, and antimutagenic activities [7,11]. These TPCs are known for the presence of one or more phenol groups, which are aromatic rings with a hydroxyl group [12]. Ersan et al. [13] reported cyanidin, quercetin, and gallic acid in the PGH. Moreno et al. [14] evaluated the Kerman variety, identifying gallic acid and catechin. In contrast, the Bronte variety contained quercetin and eriodictyol, and in the Uzun and Ohadi varieties, chlorogenic acid and luteolin were found [14]. On the other hand, Noorolahi et al. [15] reported gallic acid, quercetin, galloyl-shikimic acid, galloylquinic acid, and galloyl-*O*-hexoside.

The SSF is a viable biotechnological solution that is not only environmentally friendly but also economically profitable. This innovative approach is becoming increasingly essential for utilizing agro-industrial wastes [16]. There are studies related to the use of *Aspergillus niger*, considered a GRAS microbe (generally recognized as a safe organism) [17], in SSF with various agricultural by-products such as cascalote pods [18], pomegranate peel [19], Mexican rambutan peel [20], pineapple residue [21], mango seed [22], fig by-products [23], Castilla rose [24], and others. In these studies, the fungus has contributed to the recovery of TPC by producing enzymes that degrade the cell wall of agricultural wastes, thereby enhancing the phenolic content. This adds value to these by-products for developing new products [18,19,20,21,22,24,25,26,27]. This study aims to assess the potential of PGH as a support in SSF with *A. niger* and its impact on the release of TPC with antioxidant activity and its identification. This study can prove the potential usefulness of PGH in the consumer goods industry.

## 2. Materials and Methods

### 2.1. Preparation of the Substrate

The PGH var. Sfax (experimental population) was obtained from anonymous pistachio plots in Meoqui, Chihuahua, Mexico (28°16′04″ N 105°28′56″ O). The material was disinfected with a UV lamp (LES-04, Megaluz, China) for 1 h, sun-dried for 6 h, and then dried again in an incubator (Incubator FE-131, Felisa, Zapopan, Jalisco, Mexico) at 60 °C for 72 h [13]. After that, PGH was ground to reach a particle size of 0.85 mm and stored in jars protected from light until its use.

### 2.2. Determination of Hydrological Parameters of PGH

The critical humidity point (CHP) was determined according to Orzua et al. [28]. For that, 1 g of PGH was weighed and dried in a thermobalance (MB120, Parsippany, NJ, USA) at 105 °C. The changes in the weight and moisture of the material were recorded, and drying curves were elaborated. The water absorption index (WAI) was obtained following Torres-León et al. [22], where 1.5 g of PGH was placed in a tube (Falcon) of 50 mL with 15 mL of distilled water. The sample was mixed in a vortex (Four E’S Scientific, Guangzhou, China) for 1 min and centrifuged at 3000× *g* for 10 min. The supernatant was discarded, and the wet sample was recovered, weighed, and dried. According to Cerda-Cejudo et al. [20], the maximum moisture content (MMC) of the material was calculated using the solids balance of the PGH, moisture content, and the values obtained from the WAI.

### 2.3. Microorganism and Inoculum Production

*Aspergillus niger* GH1 strain, belonging to the culture collection of the Food Research Department of the Autonomous University of Coahuila and deposited in the Mycoteca of the University of Minho (MUM:23.16), was used. The strain was reactivated on potato-dextrose agar (PDA) (Bioxon^®^, Mexico City, Mexico) and incubated in a compact microbiological incubator (Heratherm IMC 18, Thermo Scientific, Langenselbold, Hesse, Germany) at 30 °C for 5 d. Once active, it was inoculated in Erlenmeyer flasks (250 mL) with 30 mL of PDA and incubated in a compact microbiological incubator at 30 °C for 5 d. The spores were collected with a sterile 0.01% (*v*/*v*) Tween-80 solution and counted in a Neubauer chamber [18].

### 2.4. Release of Phenolic Compounds via Solid-State Fermentation

Polypropylene-packed bed column bioreactors (PBCB) were used to evaluate the microorganism growth and biological release of phenolic compounds. The bioreactors were packed with 5 gdm of sterile PGH impregnated with 5 mL of Czapek-Dox culture medium ([KCl (1.52 g/L), KH_2_PO_4_ (3.04 g/L), MgSO_4_ (1.52 g/L), and NaNO_3_ (7.65 g/L)] and inoculated with *A. niger* GH1 (1 × 10^6^ spores/mL). During the fermentation process (72 h), PBCB were incubated at 30 °C, and forced aeration was supplied (one liter per kilogram of wet mass per minute (L/Kgwm min)). Additionally, experimental units were taken every 12 h to determine the TPC and AC. Experimental units from 0 h were considered as a control. Conversely, microbial growth and substrate consumption were indirectly estimated through CO_2_ production and O_2_ consumption [29]. At the end of the culture, the concentration of phenolic compounds and the antioxidant capacity were determined from the fermented material. These analyses require preparing an extract from the fermented material. Therefore, samples of 1 gwm from the culture bed were suspended in 5 mL of ethanol, distilled water, and lactic acid solution (80:19:1 *v*/*v*/*v*). The samples were then sonicated for 20 min and filtered through cotton and 0.45 µm membranes (Millipore™, Darmstadt, Germany). The filtered extracts were placed into 2 mL vials and stored at −18 °C until their analysis.

### 2.5. Effect of Moisture Content, Inoculum Size, and Aeration on TFC Release

Once the time of the maximum release of TPC and AC was selected, another SSF process was carried out to explore the effect of moisture (40, 50, and 60%), inoculum concentration (1 × 10^6^, 5 × 10^6^, and 1 × 10^7^ spores/mL), and aeration (0.5, 1, and 1.5 L/Kgwm min) (Table 1). The extracts were obtained after 24 h of SSF according to the described protocol in Section 2.4, and it was used to determine the TPC and AC.

### 2.6. Analytical Methods

#### 2.6.1. Respirometry Analysis

Respirometry monitoring was implemented to estimate the fungal growth and substrate consumption. A respirometric analyzer described by Méndez-González et al. [30] was used for online measurement of CO_2_ production, O_2_ consumption, and airflow. The O_2_ consumption (OCR) and CO_2_ production (CPR) rates were obtained from the concentration gradients between the bioreactor exhaust gas and the air supplied and were expressed in mg/gidm h (gidm, gram of initial dry mass). The total O_2_ consumption (TOC) and CO_2_ production (TCP) were estimated using the trapezoidal method [29] to determine the area under the OCR and CPR curves (respectively). TOC and TCP were expressed in mg/gidm.

Kinetical parameters linked to CO_2_ production and O_2_ consumption were estimated using the Logistic and Pirt models (Equations (1) and (2)) [31].

(1) $$\frac{{dCO}_{2}}{dt}={\mu CO}_{2}\left[1-\frac{{CO}_{2}}{{CO}_{2max}}\right]$$

(2) −dO2dt=1YCO2O2 dCO2dt+mO2CO2

The specific CO_2_ production rate (µ), the initial CO_2_ production (CO_2o_), and the total CO_2_ production (CO_2max_) were estimated by integrating Equation (1) into Equation (3) using the generalized gradient reduction method [30].

(3) $${CO}_{2}=\frac{{CO}_{2_{max}}}{1-\left[\left(\frac{{CO}_{2_{max}}-{CO}_{2_{0}}}{{CO}_{2_{0}}}\right)\right]\;e^{-\mu t}}$$

The Logistic and Pirt models were coupled as suggested by Méndez-González et al. [30] Equation (4) to describe the TOC curve. Equation (4) was integrated to estimate the O_2_ consumption as a function of CO_2_ production in Equation (5). The coefficients associated with the mass yield of CO_2_ per O_2_ consumption (Y_CO_2_/O_2__) and maintenance coefficient (m_O_2__) were estimated by multilinear regression.

(4) −dO2−CO2YCO2O2dCO2=mO2μ1−CO2CO2max

(5) O20−O2=1YCO2O2CO2−CO20+mO2CO2maxμLnCO2max−CO20CO2max−CO2

#### 2.6.2. Hydrolysable Phenols (HP)

To determine the HP content of the PGH, 20 µL of sample and 20 µL of Folin–Ciocalteu reagent (2 N) were placed in a 96-well microplate. After 5 min, 20 µL of a 0.01 M sodium carbonate solution was added and kept in darkness for 5 min. Finally, 125 µL of distilled water was added, and the absorbance was obtained at 790 nm in a spectrophotometer (Multiskan GO, Thermo Scientific, Vantaa, Uusimaa, Finland) [32]. The results were expressed as mg of gallic acid/g of dry mass (mgGA/gdm). A gallic acid standard curve was used (y = 1.8216x).

#### 2.6.3. Condensed Phenols (CP)

Ferric reagent and HCl–butanol (1:9 *v*/*v*) were used to determine the CP content of the PGH. 250 µL of sample, 1500 µL of HCl–butanol, and 50 µL of ferric reagent were placed in a hermetic tube. The mixture obtained was kept boiling for 40 min. Subsequently, the samples were cooled at room temperature, and 200 µL were placed in a 96-well microplate, where the absorbance at 460 nm was measured in a spectrophotometer [20]. The results were expressed as mg of catechin equivalents/g of dry mass (mgCE/gdm). A catechin standard curve was used (y = 0.6359x).

The TPC was calculated as the sum of HP and CP values and expressed as mg/g of dry mass (mg/gdm).

#### 2.6.4. Antioxidant Capacity

The antioxidant capacity of the extracts with DPPH, ABTS, and FRAP assays was evaluated. For the DPPH assay, 60 μM DPPH solution (2,2-diphenyl-1-picrylhydrazyl) (Sigma-Aldrich^®^, Naucalpan de Juarez, Mexico) in absolute methanol was prepared. 7 µL of sample and 193 µL of DPPH reagent were placed in a 96-well microplate and kept in darkness for 30 min at room temperature before measuring the absorbance at 517 nm [33]. The results were expressed as mg of Trolox equivalents/g of dry mass (mgTE/gdm). A Trolox (6-hydroxy-2,5,7,8-tetramethylchroman-2-carboxylic acid) standard curve was used (y = 2.2641x).

For the ABTS assay, 7 mM ABTS solution (2,2-azinobis-(3-ethylbenzothiazoline-6-sulfonate) (Sigma-Aldrich^®^) in absolute ethanol was prepared and mixed with a 2 mM potassium persulfate solution in a 2:1 *v*/*v* ratio. This solution was kept at room temperature in the dark for 12 to 16 h. For the assay, 10 µL of sample and 190 µL of ABTS reagent were placed in a 96-well microplate, and the absorbance was recorded at 734 nm [34]. The results were expressed as mg of Trolox/g of dry mass (mgTE/gdm). A Trolox (6-hydroxy-2,5,7,8-tetramethylchroman-2-carboxylic acid) standard curve was used (y = 3.4308x).

To determine the iron-reducing antioxidant power (FRAP) of PGH, a 0.3 M acetate buffer solution at pH 3.6, a 10 Mm TPTZ solution (2,4,6-tri(2-pyridyl)-s-triazine (Sigma-Aldrich^®^)) in 40 mM HCl and a 20 mM FeCl_3_*6H_2_O solution were prepared. Then, the solutions were mixed in a 10:1:1 (*v*/*v*/*v*) ratio, and the FRAP reagent was incubated at 37 °C for 30 min. For the assay, 6 µL of sample, 18 µL of distilled water, and 180 µL of FRAP reagent were placed in a 96-well microplate and incubated at 37 °C for 1 h [18]. The absorbance was determined at 593 nm, and results were expressed as mg of Fe^2+^/g of dry mass (mgFe^2+^/gdm). A Fe_2_SO_4_ standard curve (Sigma-Aldrich^®^) was used (y = 7.5781x).

#### 2.6.5. Identification of Phenolic Compounds by RP-HPLC–ESI-MS

The extract obtained was purified by column chromatography using Amberlite XAD-16 (Sigma-Aldrich^®^, St. Louis, MO, USA), according to the report of Ascacio-Valdés et al. [35]. The solvent was removed by evaporation until a powder was obtained, which was used to determine the profile of TPC present through RP-HPLC–ESI-MS analysis. Reversed-phase high-performance liquid chromatography analysis was performed according to the methodology used by Cerda-Cejudo et al. [20] on a Varian HPLC system that includes an autosampler (Varian ProStar 410, Palo Alto, CA, USA), a ternary bomb (Varian ProStar 230I, Palo Alto, CA, USA), and a photodiode array detector (Varian ProStar 330, Palo Alto, CA, USA). The HPLC system, coupled with an ion trap mass spectrometer (Varian 500-MS IT Mass Spectrometer, Palo Alto, CA, USA) and an electrospray ion source, was also used, operating in negative mode [M-H]^−1^. MS Workstation software (V 6.9) was used to collect data and process, in full-scan mode, acquired in the m/z range of 50–2000.

### 2.7. Statistical Analysis

A completely randomized design was established to determine the effect of fermentation time on the TPC content (HP + CP) and AC (DPPH, ABTS, and FRAP) of the fermented PGH. Seven-time levels were evaluated (0, 12, 24, 36, 48, 60, and 72 h), and three repetitions were performed per treatment.

Later, a completely randomized design with a 3^k^ factorial arrangement was used to evaluate the effect of moisture (40, 50, and 60%), inoculum concentration (1 × 10^6^, 5 × 10^6^, and 1 × 10^7^ sp/mL), and aeration rate (0.5, 1, and 1.5 L/Kgwm min) on the AC (DPPH, ABTS, and FRAP) and TPC content. The STATISTICA 7.0 program obtained a Box-Behnken 3^k^ experimental matrix with 15 treatments (Table 1). Three repetitions were carried out per treatment.

The data obtained were analyzed using an analysis of variance (ANOVA, α ≤ 0.05), and subsequently, Fisher’s Least Significant Difference (LSD) test (α ≤ 0.05) was performed. A Pearson correlation analysis was performed between the response variables of TPC and AC (ABTS, DPPH, and FRAP). The results were examined using SAS 9.0 statistical software.

## 3. Results

### 3.1. Hydrological Parameters of Pistachio Green Hull

Each organic support presents several physicochemical parameters, so correct characterization is essential to evaluate its potential for use in an SSF process [36]. Table 2 presents the results obtained from WAI, CHP, and MMC of the PGH.

### 3.2. Release of Phenolic Compounds via Solid-State Fermentation

**Figure 1:**
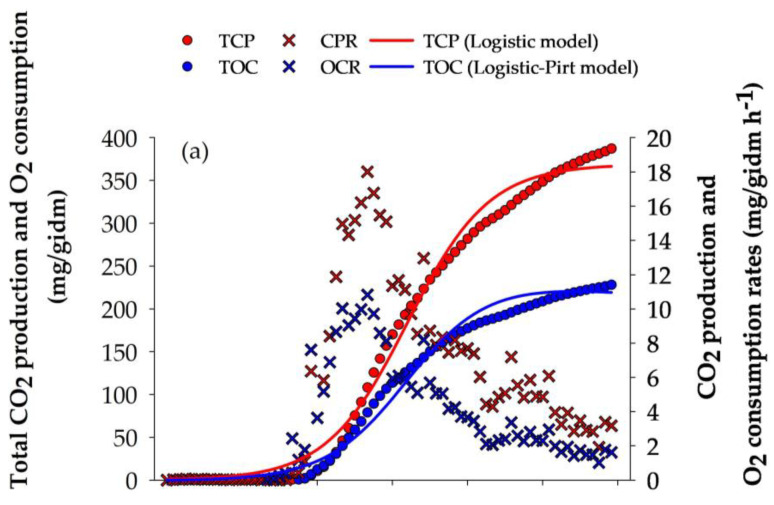

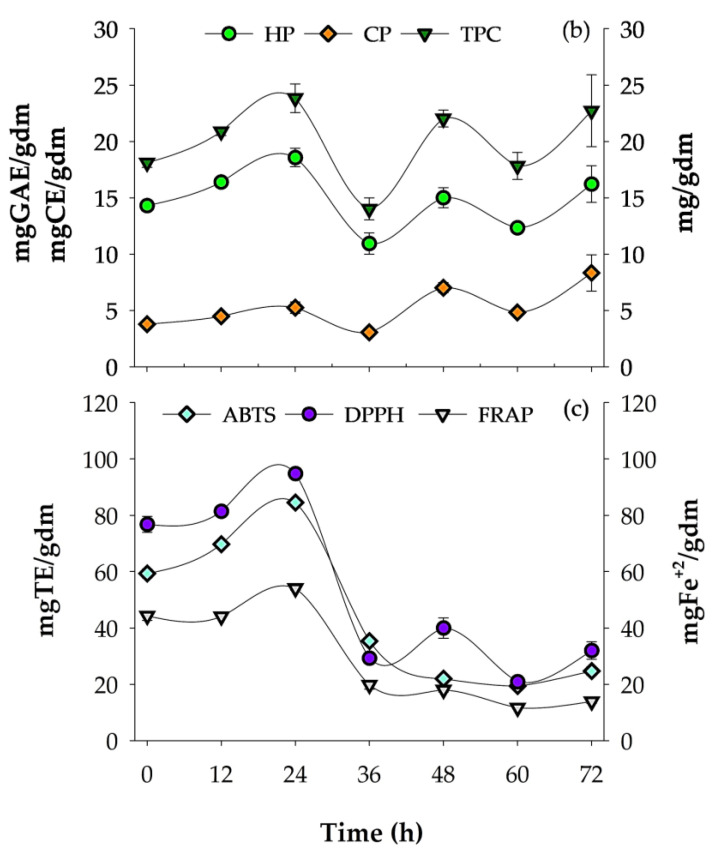
Respirometry profile of *A. niger* GH1, kinetics of phenolic compounds, and antioxidant activity during SSF in PGH. (**a**) O_2_ consumption rate (OCR) and CO_2_ production rate (CPR); total O_2_ consumption (TOC) and total CO_2_ production (TCP). (**b**) Hydrolyzable phenols (HP), condensed phenols (CP), and total phenolic content (TPC). (**c**) Antioxidant assays: ABTS, DPPH, and FRAP. gdm = gram of dry mass; mgGAE = milligram of gallic acid equivalents; mgCE = milligram of catechin equivalents; gdm = gram of dry mass; mgTE = milligrams of Trolox equivalents; mgFe^2+^ = milligram of ferrous ion; gidm = grams of initial dry mass; results are the mean (n = 3) ± standard deviation.

The evolution of the fermentation process was monitored using respirometry analysis. The maximum CPR (18.011 mg/gidm h^−1^) and OCR (10.825 mg/gidm h^−1^) were obtained at 32 h of culture (Figure 1a). At the end of the culture, TCP and TOC obtained by *A. niger* GH1 reached values of 363.08 and 217.55 mg/gidm, respectively. The TCP and TOC curves were described using Equations (3) and (5) (Figure 1a), reaching a high goodness of fit (R^2^ > 0.98) (Table 3).

During the culture, the release of phenolic compounds and antioxidant capacity were determined using HP, CP, TPC, ABTS, DPPH, and FRAP methods (Figure 1b,c). The highest content of HP and TPC was obtained at 24 h of fermentation with values of 18.57 ± 0.83 mgGAE/gdm and 23.83 ± 1.27 mg/gdm, respectively, while the highest CP content was obtained after 72 h of fermentation (8.34 ± 1.61 mgCE/gdm). The HP and TPC content after 24 h of fermentation compared to the unfermented control showed an increase of 29.76% and 38.42%, respectively; additionally, the TPC content at 24 h of cultivation was 4.88% higher than the 72 h time, which was the second value with the highest TPC content. In contrast, the 36 h time was the lowest phenolic content presented, with a percentage of 70% lower than obtained at 24 h.

Regarding the AC of the fermented PGH extracts, the highest result was observed after 24 h of culture, with values of 84.48 ± 0.30 mgTE/gdm, 94.79 ± 0.89 mgTE/gdm, and 54.02 ± 0.62 mgFe^2+^/gdm for the DPPH, ABTS, and FRAP assays, respectively. Compared to the unfermented treatment, increases of 42.43%, 23.42%, and 21.96% were observed after 24 h of SSF for the ABTS, DPPH, and FRAP assays, respectively.

The Pearson correlations obtained for the different response variables of phenolic content and AC are listed in Table 4. The results show that there is a consistent positive linear relationship between the HP content and AC for the three measurement methods (DPPH, ABTS, and FRAP) (R ≤ 0.6902), and these findings are statistically significant (α ≤ 0.05). This suggests that as the HP content increases, the AC also increases.

### 3.3. Effect of Moisture Content, Inoculum Size, and Aeration on TPC Release

The results obtained for phenolic content and AC are presented in Table 5. Results demonstrate an increase in the phenolic content in all treatments compared to the unfermented control. Treatment 8, with conditions of 60% moisture, 1 × 10^7^ sp/mL, and 1 L/Kgwm min of aeration, yielded the best results with values of 34.71 ± 1.04 mgGAE/gdm, 6.77 ± 0.28 mgCE/gdm, and 41.48 ± 0.99 mg/gdm for HP, CP, and TPC, respectively. The TPC of treatment 8 increased by 129% compared to the unfermented control. Treatment 1, which had 60% moisture, 5 × 10^6^ sp/mL, and 0.5 L/Kgwm min of aeration, was the second-best treatment, with a TPC value of 37.03 ± 2.21 mg/gdm. When compared to treatment 8, there was only a 12% difference; in contrast, treatment 7, with 40% moisture, 1x10^7^ sp/mL, and 1 L/Kgwm min of aeration, showed the smallest increase, with a TPC value of 28.58 ± 0.44 mg/gdm, which was 45% lower than treatment 8.

Due to the strong correlation between TPC and AC, the highest increase was also observed in treatment 8, with 132.75 ± 4.91 mgTE/gdm and 131.68 ± 2.49 mgTE/gdm for DPPH and ABTS, respectively. In the case of the FRAP assay, no significant differences (*p* ≤ 0.05) were observed between treatments 1 and 8, with values of 532.96 ± 29.98 and 504.59 ± 8.71 mgFe^2+^E/gdm, respectively. Therefore, treatment 8 allowed the highest increase of AC in the three methods, with increases of 124, 71, and 1039% (ABTS, DPPH, and FRAP, in that order), compared to the unfermented control.

Table 6 displays the impact of the factors above on the release of TPC, HP, CP, and AC. Moisture content positively influenced all response variables, and this effect was statistically significant (α ≥ 0.05). The inoculum concentration had a significant positive effect on half of the response variables (α ≥ 0.05). Increasing the inoculum concentration was found to favor the content of HP, TPC, and the AC in DPPH radical, while it had no significant effect on the AC in ABTS and FRAP assays, as well as the CP content. Aeration during the SSF process had a significant negative effect (α ≥ 0.05) on all response variables except AC (ABTS). An increase in the aeration rate reduced TPC, HP, CP, and AC (DPPH and FRAP).

**Figure 2:**
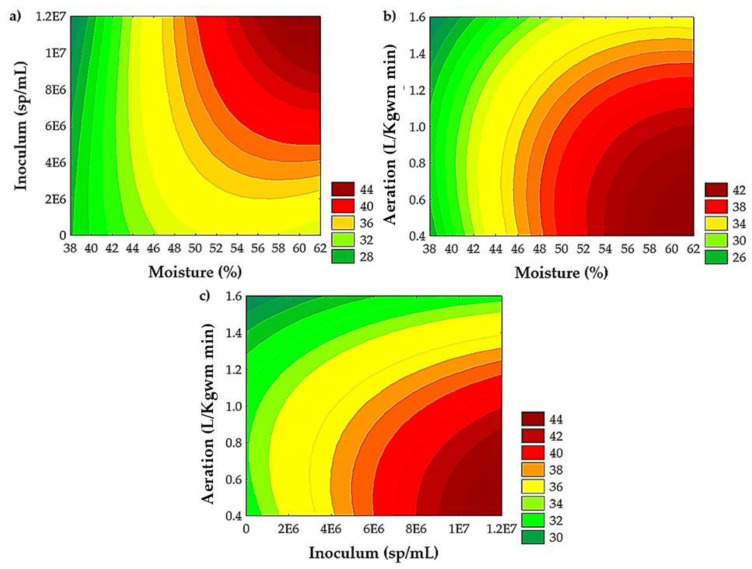
Interactive effects of (**a**) moisture and inoculum, (**b**) moisture and aeration, and (**c**) inoculum and aeration on yield extraction of TPC from PGH by SSF with *A. niger* GH1.

Figure 2 shows the contour plots to analyze the combined effects of factors on the extraction yield of TPC. The contour plots showed an improvement in yield extraction of TPC, ranging from 56 to 62% of moisture (Figure 2a,b), with 0.4 to 0.5 L/Kgwm min of aeration (Figure 2b,c) and an inoculum of 9 × 10^6^ to 1.2 × 10^7^ sp/mL (Figure 2a,c).

### 3.4. Identification of Phenolic Compounds by RP-HPLC–ESI-MS

A water-soluble fraction and an oily fraction were found. The HPLC–MS analyzed the water-soluble fraction, and two masses (330.9 and 950.5 *m*/*z*) corresponding to gallic acid 4-*O*-glucoside and geraniin were detected (Table 7).

## 4. Discussion

Since water is essential for the transport of nutrients from the substrate to the cells, hydrological parameters such as CHP, WAI, and MMC were determined. The CHP indicates the amount of bound water in the solid matrix, which is not available for the microorganism [28,37,38]. Therefore, a high CHP could affect the growth of the microorganism [18,22]. For SSF, values of CHP lower than 40% are recommended for *Aspergillus niger* [37]. The PGH obtained a CHP of 0.37 ± 0.05%, which indicates that the most water content in the solid substrate is available for the culture. On the other hand, WAI refers to the water absorption capacity of a solid matrix, which is linked to hydrophilic groups in its molecular composition [22]. WAI values from 2.97 to 12.09 g gel/gdm facilitate microbial growth in solid-state substrates [37]. The PGH obtained a WAI value of 5.73 ± 1.05 g gel/gdm, which indicates a proper water absorption capacity. In fact, this value is higher than those obtained in other materials used in SSF processes, such as cascalote pods (2.97 ± 0.07 g gel/gdm) [18], rambutan peel (3.4 ± 0.01 g gel/gdm) [20], Mexican mango seed (3.5 ± 0.04 g gel/gdm) [22], and fig residues (3.74 ± 0.10 g gel/gdm) [23]. The MMC is essential to determine the initial conditions for SSF. Water excess can affect the gas exchange and cause particle agglomeration [20]. The PGH presents a MMC of 82.79 ± 2.77%. The values obtained are higher than those reported for cascalote pods (79.33 ± 2.08%) [18] and rambutan peel (72 ± 0.03%) [20]. García-Zapata [39] indicates that fungi need a moisture content between 40 and 60%, so the PGH provides optimal water conditions to *A. niger* GH1. Due to the hydrological characteristics of PGH, this waste is suitable for use in SSF to release phenolic compounds.

To verify the above, *A. niger* GH1 was inoculated on pretreated PGH to phenolic compounds via SSF. During the fermentation process, CO_2_ production and O_2_ consumption were monitored to estimate the culture evolution. The maximum value of CPR and OCR reached by *A. niger* (Figure 1a) was obtained at 32 h, which is comparable with those obtained in other substrates [40,41]. Therefore, the substrate composition does not limit the *A. niger* growth. In fact, the term of Equation (5) associated with Y_CO_2_/O_2__ is higher than that associated with m_O_2__, so biomass production was favored during the culture [30,31,42]. However, the maximal phenolic compound release was obtained at 24 h, near the beginning of the exponential growth phase. In this stage, the energy from substrate consumption is directed equitably to microbial growth and cellular maintenance, favoring enzyme production [43].

During SSF, a wide range of enzymes is produced, leading to structural modifications caused by the biotransformation of the cell wall. This process also results in changes to the organic compounds, such as an increase in protein content and a reduction in carbohydrate and fatty acid content. As a result, this facilitates the release of nutrients and bioactive molecules, including phenolic compounds, which are associated with the structural components of the plant matrix [44].

As mentioned, the SSF process with *A. niger* GH1 presented the highest phenolic content and AC after 24 h of culture (Figure 1b,c), and it has been reported that this microorganism has a short adaptation phase, reducing the time of maximum growth and the production of some metabolite [28]. The use of *A. niger* GH1 in SSF processes has been reported to recover TPC with AC from different wastes such as rambutan peel [20], Mexican mango seed [22], Castilla rose [24], and pineapple residues [21], with the maximum release of TPC at 12, 20, 24, and 30 h of culture, respectively. From these data, it is possible to establish a range of 12 to 30 h as the time in which *A. niger* GH1 releases the highest amount of TPC with AC in SSF processes using agro-industrial wastes as a substrate.

The time with the highest AC coincides with the highest phenolic content, which confirms that these compounds released in PGH increase the AC. As evidence of this, the Pearson correlation coefficient (Table 4) indicates that there is a regular positive linear relationship between the HP content and the AC for the three measured methods (DPPH, ABTS, and FRAP) (r ≤ 0.6902). These values were statistically significant (α ≤ 0.05), which indicates that increasing the HP content (corresponding to the majority fraction observed in Table 3) also increases AC. On the other hand, the Pearson correlation coefficient of CP content and AC showed a weak negative linear relationship (r ≤ −0.3634). This can be explained due to HP stands out for its antioxidant properties, while CP for its bacteriostatic and fungicidal activity thanks to their structure [45], however, Vázquez-Flores et al. [46] mentioned the antioxidant activity of both groups.

The PGH contains a higher content of HP than CP in all treatments (Table 5). This can be explained by the structure of the PGH, which, being an agro-industrial waste, presents a large number of organic molecules such as pectin or structural carbohydrates such as cellulose, hemicelluloses, or lignin [25]. For this reason, there is a higher fraction of TPC bound with these organic compounds than those that create flavonoid polymers such as CP [46].

The SSF process of the PGH showed treatment 8 (60% moisture, 1 × 10^7^ sp/mL, and 1 L/Kgwm min of aeration) with the highest TPC, HP, CP, and AC (Table 5). Compared to the other studies, only two reports of SSF processes use PGH as a substrate. Abbasi et al. [47] performed an SSF at 30 °C and 70% moisture for 20 days, using the fungus *Phanerochaete chrysosporium*. The maximum TPC was obtained after 16 days [63 mgCAE/gdm (Caffeic acid equivalents)], representing an increase of 29% compared to the control. Although they are not the same units, the value is higher than the 41.48 mgGAE/gdm obtained in this project. However, the authors report the addition of soybean as a nitrogen source in the culture medium, which may overestimate the value of TPC since soybean contains 10.3 to 13.7 mg/g [48]. Karimi et al. [49] reported the SSF of PGH for 10 days at 30 °C and 66% initial moisture, using the fungus *A. terreus* ATCC74135 (1 × 10^6^ sp/gdm), where a decrease of 81.5% in the TPC was obtained, compared to the unfermented control, attributed to possible hydrolysis when using 6 M HCl during the extraction or to the consumption of the TPC by the fungus due to long-term fermentation. The carbon source is consumed, and the microorganism can secrete enzymes capable of degrading TPC and use them as a carbon source to perform its metabolic actions [22]. On the other hand, studies have reported the extraction of TPC from PGH using various extraction techniques, with values from 22.2 to 127.25 mg/gdm [2,8,11,15,50,51,52]. For these studies, the differences in phenolic content are attributed to the extraction method, the solvent used, time, temperature, solid/solvent ratio, crop, geographical location, harvest season, or the pistachio variety [2,8,50]. However, the results obtained in this project are superior to those reported by Noorolahi et al. [15], Erşan et al. [51], and Neval-Özbek et al. [11], demonstrating the effectiveness of the bioprocess as a strategy to increase the TPC content from PGH.

Several studies have demonstrated the positive effect of SSF with *A. niger* GH1 on the TPC from various plant substrates. De León-Medina et al. [24] reported a 166% increase in TPC in extracts fermented by *A. niger* GH1 from rosa Castilla. Torres-León et al. [22] increased the TPC content by 230% in Mexican mango seeds using *A. niger* GH1. During the SSF process of Mexican rambutan peel with *A. niger* GH1, Cerda-Cejudo et al. [20] obtained an increase in TPC content of 404%. López-Cárdenas et al. [18] increased the TPC content by 219% in ground cascalote pods using *A. niger* GH1; Buenrostro-Figueroa et al. [19] achieved an increase of ~526% in pomegranate peel using *A. niger* GH1; and Ramírez-Esparza et al. [53] increased the TPC content by 83% in corn with the fungus *R. oryzae*. Using the *A. niger* HT4 strain in SSF from fig residues, Buenrostro-Figueroa et al. [23] reported an increase in TPC of 548%. In the case of PGH, the TPC of treatment 8 increased by 129% compared to the unfermented control. The increase in phenolic content is because, during the SSF process, the microorganism produces enzymes such as cellulases, lipases, proteases, and amylases responsible for the degradation of cell wall components, thus releasing the bound fraction of TPC, which passes into the free fraction, improving its solubility and extractability [18,19,20,21,22,24,26,27,53,54].

Due to the strong correlation between TPC and AC, the highest increase was also observed in treatment 8, with 132.75 ± 4.91 mgTE/gdm and 131.68 ± 2.49 mgTE/gdm for DPPH and ABTS, respectively. In the case of the AC of the FRAP assay, no significant differences (*p* ≤ 0.05) were observed between treatment 1 and 8, with values of 532.96 ± 29.98 and 504.59 ± 8.71 mgFe^2+^/gdm, respectively. Therefore, treatment 8 allowed the highest increase in AC in the three methods, with increases of 124, 71, and 1039% (ABTS, DPPH, and FRAP, in that order), compared to the unfermented control. The increase in AC is attributed to the fact that treatment 8 presented the highest content of TPC (Table 5), which increases said biological properties due to its antioxidant nature [46]. However, as was noted in the present study, a decrease in the AC of fermented extracts of PGH against the DPPH radical has also been reported in another study. During SSF with *A. terreus* ATCC74135, Karimi et al. [49] observed a 9% decrease after 10 days of culture. On the other hand, Abbasi et al. [47] found that by subjecting the PGH to SSF with *P. chrysosporium* for 16 days, there was a 22% reduction in AC. This decrease can be attributed to the biodegradation or biotransformation of TPC, which leads to their consumption or modification, thus affecting AC [22].

Studies analyzed the AC of the PGH, with values of 127.65 to 411.98 mgTE/gdm, 117.64 to 466.73 mgTE/gdm, and 122.64 to 2230.8 mgFe^2+^E/gms for DPPH, ABTS, and FRAP, respectively [15,51]. However, the results obtained in this project are superior to those reported by Erşan et al. [51], demonstrating the effectiveness of the bioprocess as a strategy to increase TPC with AC of the PGH.

Previous studies have reported that SSF increases the AC in extracts obtained from several plant matrices. López-Cárdenas et al. [18] increased the AC by 66.76, 93, and 55.67% for DPPH, ABTS, and FRAP, respectively, in cascalote pods. Buenrostro-Figueroa et al. [19] achieved an increase of 581% for DPPH in pomegranate peel. Recently, Ramírez-Esparza et al. [53] reported an increase of 55, 119, and 125% (ABTS, DPPH, and FRAP, respectively) in corn.

Moisture content showed a positive effect for all response variables, where increasing moisture in the SSF process will also increase TPC, HP, CP, and AC (Table 6). It is known that the different microbial species studied in SSF processes require different moisture contents to achieve their development and a correct release of TPC [19,55]. In SSF processes with fungi, a moisture content of 20 to 70% is recommended [16], while values above 70% cause difficulty in the growth of the microorganism and the production of enzymes, as well as the physical properties of the substrate by higher agglomeration of particles and limitation of oxygen transfer [18,24,28]. On the other hand, moisture values below 20% induce sporulation [36], and this is due to the small amount of available water found in the medium, so the microorganism seeks to perpetuate itself and stop growing until moisture conditions are favorable again to continue with their growth and development [56].

The effect of moisture on the growth of *A. niger* GH1 and the recovery of bioactive compounds has been reported. López-Cárdenas et al. [18] evaluated the SSF process of cascalote pods at two moisture levels (50 and 60%). A positive effect was observed, with higher achievement of TPC and AC at 60%. On the other hand, Cerda-Cejudo et al. [20] evaluated the effect of moisture (50, 60, and 70%) in an SSF process with *A. niger* GH1 on Mexican rambutan peel. The authors observed a positive quadratic effect: the increase in moisture content favors the recovery of ellagic acid to a certain extent, reaching the maximum value at 60% moisture, and above this (70%), the value decreases. By evaluating two levels of moisture (50 and 60%), Buenrostro et al. [19] observed an opposite effect during the SSF with *A. niger* GH1 on pomegranate peels, establishing 50% moisture as the optimal value for the release of the TPC. The studies by López-Cárdenas et al. [18] and Cerda-Cejudo et al. [20] agree with the results obtained in the present study, where a moisture content of 60% was obtained using *A. niger* GH1 as in the previously mentioned studies. This moisture content of 60% is the value required for the SSF process of the PGH (Figure 2a,b). Because each plant material has a different physicochemical composition, the moisture conditions used for each bioprocess will differ [57].

The inoculum concentration showed a positive effect where increasing the inoculum concentration favors the content of HP, TPC, and AC (the DPPH radical) (Table 6). Being an important parameter during an SSF process, the inoculum has been widely studied since the success of the process depends on it. The adequate concentration of inoculum allows the reduction of the time of the adaptation phase of the microorganism, accelerating microbial growth and the production of secondary metabolites and reducing fermentation times to obtain products of interest [36]. The use of filamentous fungi is attributed to their ability to adapt to a wide variety of substrates and carbon sources, thanks to the hydrolytic enzymes that they produce, and additionally contribute to the release or bioconversion of TPC [18].

The increase in inoculum concentration promotes a higher release of TPC through SSF with *A. niger* GH1 on rambutan peel, Castilla rose, and cascalote pods [18,20,24]. The results shown for the *A. niger* GH1 strain demonstrate higher efficiency in the release of TPC using high concentrations of inoculum (Figure 2a,c), which translates into greater generation or synthesis of enzymes that release these compounds [27].

Aeration during the SSF process had a significant negative effect on all response variables except for ABTS, whereby a reduction in TPC, HP, CP, and AC (DPPH and FRAP assays) was observed when increasing aeration (Table 6). Aeration plays an important role in the removal of heat and CO_2_ from the medium, which are products of microbial metabolism. Likewise, it supplies the oxygen required by the microorganism for its growth and development [58,59,60]. There are no reports of the effect of aeration in an SSF process on TPC release. However, its effect on biomass production and various enzymes has been evaluated. Méndez-González et al. [29] evaluated five aeration levels (0.16, 0.33, 0.66, 0.96, and 1.28 L/Kgwm min) on the production of *Metarhizium robertsii* conidia, obtaining the highest value using the lowest level (0.33 L/Kgwm min). Ridder et al. [61] reported the production of xylanases from *Trichoderma longibrachiatum* at three aeration levels (0, 2.9, and 5.7 L/Kgwm min), with the highest enzymatic activity at 2.9 L/Kgwm min. On the other hand, Zhou et al. [60] conclude that the glycoprotein production of *Streptomyces kanasenisi* ZX01 increases as aeration increases, with the highest value at an aeration rate of 2.0 vvm (volume of air per liter per minute, similar to L/Kgwm min). According to the above, each SSF process is unique, and therefore, the aeration rate will depend on the product obtained, the substrate, and the microorganism used. In the case of PGH, this decrease in TPC (Figure 2b,c) and AC observed in SSF can be explained by the possible capture of free radicals given by the TPC generated by the SSF process [62].

Based on the results obtained, treatment 8 presents the best performance in terms of TPC recovery from PGH by SSF with *A. niger* GH1 compared to the unfermented control, where an increase of 129, 142, and 78% is reflected for the response variables TPC, HP, and CP, respectively. Regarding the AC obtained after the SSF process, an increase of 72, 124, and 1.039% was obtained for the response variables DPPH, ABTS, and FRAP, respectively. In general, the SSF process of PGH with *A. niger* GH1 requires high moisture conditions (60%), a high inoculum concentration (1 × 10^7^ sp/mL), and a reduced aeration rate (0.5 L/Kgwm min) to enhance the performance of TPC with AC in the PGH.

The TPC profile showed gallic acid 4-*O*-glucoside, which has already been widely reported, since Moreno-Rojas et al. [14], Arjeh et al. [9], Ghandahari et al. [63], and Grace et al. [52] report it as one of the majority TPC in the PGH. This compound has the lowest molecular weight of TPC and presents antioxidant activity (by being a donor of electrons or hydrogen atoms) and antibacterial activity by increasing the permeability of membranes in bacteria [9,63] and the changes it causes in pH [8]. The other compound shown by the profile was geraniin, which also presents antioxidant activity thanks to its ability to capture radicals; anticancer potential since it prevents the proliferation of cancer cells; cytoprotective activity by generating a strong barrier that can protect the different tissues of the human; and antibacterial activity [64]. These compounds obtained are responsible for the AC observed in the PGH extracts. However, an oily fraction was also obtained, and Arjeh et al. [9], Grace et al. [52], and Schulze et al. [65] reported the presence of phenolic lipids in PGH, which have an aromatic ring with a long aliphatic chain, which gives them biological properties. These phenolic lipids are known as anacardic acids, and it is probable that these compounds were the oily fraction obtained during chromatography but were not analyzed. These compounds are classified within phenolic acids and are a taxonomic character since they are only present in plant species belonging to the Anacardiaceae family, such as pistachio [9].

Phenolic compounds have a range of applications across different industries. In the pharmaceutical sector, these compounds are widely used as antioxidants due to their ability to donate hydrogen atoms or electrons to free radicals, thereby halting oxidation reactions and potentially inhibiting health issues such as cancer, hypertension, and diabetes. In the cosmetic industry, phenolic compounds are incorporated into various products as bioactive ingredients. Their structure enables them to capture ultraviolet radiation through chromophores, protecting the skin from damage caused by solar exposure. In the textile industry, phenolic compounds serve as natural dyes, helping to reduce water pollution associated with synthetic dyes while also providing solar protection for cotton garments. In the food industry, these compounds are utilized as colorants and additives, enhancing food preservation thanks to their antioxidant and antimicrobial properties, which can extend shelf life. However, it is essential to examine the bioavailability and absorption of these compounds in vivo, as these factors can influence their bioactive properties [66].

## 5. Conclusions

This study showed that PGH could be used as a substrate in SSF due to its high moisture content (82.29%) and water absorption capacity (5.73 g gel/gdm).

*Aspergillus niger* GH1 was able to grow and invade the PGH, showing the highest release of TPC and AC at 24 h of fermentation with the process conditions of 60% moisture, an inoculum concentration of 1 × 10^7^ sp/mL, and 1 L/Kgwm min of aeration.

The SSF process significantly increased the TPC, ABTS, DPPH, and FRAP in PGH by 129, 71, 124, and 1039%, respectively.

The purified PGH extract contains gallic acid 4-*O*-glucoside and geranine, an important bioactive compound with antioxidant and antimicrobial properties. This study demonstrates the antioxidant potential of PGH after SSF, which constitutes a biotechnological strategy to valorize agroindustry residues in order to recover molecules with potential applications in the consumer goods industry, such as the food industry, cosmetics, or pharmaceuticals.

## Figures and Tables

**Table 1:** Treatment matrix of the Box-Behnken 3^k^ experimental design.

Factor	−1	0	1
Moisture (%)	40	50	60
Inoculum (sp/mL)	1 × 10^6^	5 × 10^6^	1 × 10^7^
Aeration (L/Kgwm min)	0.5	1	1.5
Treatment	Moisture	Inoculum	Aeration
1	1	0	−1
2	−1	0	−1
3	0	−1	−1
4	0	1	−1
5	−1	−1	0
6	1	−1	0
7	−1	1	0
8	1	1	0
9	−1	0	1
10	1	0	1
11	0	−1	1
12	0	1	1
13	0	0	0
14	0	0	0
15	0	0	0

L/Kgwm min = liter of air per kilogram of wet mass per minute; sp/mL = spores per mL.

**Table 2:** Physicochemical characterization of pistachio green hull.

Parameters	Results
Critical humidity point (%)	0.37 ± 0.05
Water absorption index (g gel/gdm)	5.73 ± 1.05
Maximum moisture (%)	82.79 ± 2.77

g gel = grams of gel; gdm = grams of dry mass. Results are the mean (n = 3) ± standard deviation.

**Table 3:** Respirometric parameters of *A. niger* GH1 in SSF of PGH.

Respirometric Parameter	Results
*CO_2_ production (Logistic model)*	
µ (h^−1^)	0.154
CO_2o_ (mg/gidm)	0.96
CO_2max_ (mg/gidm)	363.08
R^2^ _adj_	0.99
RSS	17,069.08
*O_2_ consumption (Logistic-Pirt model)*	
O_2o_ (mg/gidm)	0.10
O_2max_ (mg/gidm)	217.55
Y_CO_2_/O_2__ (mg_O_2__/mg_CO_2__)	1.53 ± 0.69
m_O_2_ CO_2max__/µ (mg_O_2__/mg_CO_2__ h^−1^)	3.86 ± −0.17
m_O_2__ (mg_O_2__/mg_CO_2__ h^−1^)	0.0016
R^2^ _adj_	0.99
RSS	7139.48

µ = specific CO_2_ production rate; CO_2O_ = initial CO_2_ production; CO_2max_ = total CO_2_ production; RSS = residual square sum; O_2o_ = initial O_2_ consumption; O_2max_ = total O_2_ consumption; Y_CO2/O2_ = coefficient associated with the mass yield of CO_2_ per O_2_ consumption; m_O_2__ = maintenance coefficient; m_O_2_ CO_2__/µ = coefficient associated to cellular maintenance; mg/gidm = milligram per gram of initial dry mass; mg_O_2__/mg_CO_2__ = milligram of O_2_ per milligram of CO_2_.

**Table 4:** Pearson correlation coefficient between the response variables of phenolic content (HP, CP, and TPC) and AC (DPPH, ABTS, and FRAP).

Response	HP	CP	TPC	DPPH	ABTS	FRAP
HP	-	0.4811 *	0.9058 *	0.6902 *	0.5929 *	0.5881 *
CP		-	0.8071 *	−0.2210	−0.3634	−0.3580
TPC			-	0.3579	0.2236	0.2230
DPPH				-	0.9600 *	0.9839 *
ABTS					-	0.9807 *
FRAP						-

* = significant (α ≤ 0.05); HP = hydrolysable phenols; CP = condensed phenols; TPC = total phenolic compounds; the results correspond to three replications.

**Table 5:** Phenolic content (HP, CP, and TPC) and AC (ABTS, DPPH, and FRAP) in PGH extracts subjected to SSF with *A. niger* GH1.

Treatment	HP (mgGAE/gdm)	CP (mgCE/gdm)	TPC (mg/gms)	ABTS (mgTE/gdm)	DPPH (mgTE/gdm)	FRAP (mgFe^2+^/gdm)
Control	14.31 ± 0.31 ^f^	3.80 ± 0.16 ^f^	18.11 ± 0.41 ^h^	59.31 ± 1.59 ^h^	76.80 ± 2.83 ^gh^	44.29 ± 1.59 ^j^
T1	30.16 ± 1.65 ^b^	6.87 ± 0.59 ^a^	37.03 ± 2.21 ^b^	110.03 ± 5.43 ^c^	104.20 ± 0.87 ^c^	532.96 ± 29.98 ^a^
T2	24.88 ± 1.30 ^de^	4.66 ± 0.39 ^e^	29.54 ± 1.65 ^fg^	91.65 ± 1.98 ^ef^	82.89 ± 3.41 ^f^	424.01 ± 24.74 ^bc^
T3	28.10 ± 0.84 ^c^	6.61 ± 0.03 ^a^	34.71 ± 0.84 ^cd^	98.37 ± 6.20 ^d^	83.94 ± 7.79 ^f^	385.70 ± 10.58 ^de^
T4	31.03 ± 0.06 ^b^	5.78 ± 0.02 ^b^	36.81 ± 0.05 ^b^	95.09 ± 7.05 ^de^	113.43 ± 3.18 ^b^	355.63 ± 2.30 ^ef^
T5	26.04 ± 0.52 ^de^	4.20 ± 0.30 ^f^	30.24 ± 0.24 ^efg^	83.39 ± 4.16 ^g^	85.84 ± 2.76 ^ef^	273.25 ± 22.02 ^i^
T6	27.69 ± 1.19 ^c^	5.61 ± 0.32 ^bc^	33.30 ± 1.50 ^d^	118.90 ± 2.79 ^b^	107.97 ± 4.46 ^c^	427.83 ± 13.38 ^bc^
T7	24.79 ± 0.35 ^de^	3.78 ± 0.18 ^f^	28.58 ± 0.44 ^g^	91.84 ± 4.45 ^def^	89.74 ± 1.29 ^e^	288.28 ± 19.06 ^hi^
T8	34.71 ± 1.04 ^a^	6.77 ± 0.28 ^a^	41.48 ± 0.99 ^a^	132.75 ± 4.91 ^a^	131.68 ± 2.49 ^a^	504.59 ± 8.71 ^a^
T9	26.13 ± 0.37 ^d^	4.05 ± 0.34 ^f^	30.19 ± 0.57 ^efg^	96.50 ± 0.79 ^de^	85.34 ± 2.24 ^ef^	326.47 ± 4.83 ^fg^
T10	24.91 ± 0.29 ^de^	6.49 ± 0.25 ^a^	31.40 ± 0.51 ^e^	88.13 ± 0.68 ^fg^	76.25 ± 2.43 ^gh^	314.48 ± 18.94 ^gh^
T11	28.53 ± 1.05 ^c^	4.82 ± 0.15 ^de^	33.35 ± 1.19 ^d^	116.09 ± 3.38 ^bc^	97.24 ± 1.29 ^d^	411.80 ± 33.53 ^cd^
T12	24.64 ± 0.96 ^e^	5.83 ± 0.20 ^b^	30.47 ± 0.94 ^ef^	91.18 ± 3.02 ^ef^	71.29 ± 2.91 ^h^	328.71 ± 2.77 ^fg^
T13	31.02 ± 0.72 ^b^	5.21 ± 0.04 ^cd^	36.23 ± 0.69 ^bc^	113.86 ± 3.02 ^bc^	82.39 ± 0.87 ^f^	441.93 ± 33.27 ^bc^
T14	30.70 ± 0.43 ^b^	4.67 ± 0.14 ^e^	35.37 ± 0.31 ^bc^	112.91 ± 3.72 ^bc^	83.86 ± 1.86 ^f^	429.75 ± 3.72 ^bc^
T15	30.83 ± 0.04 ^b^	4.98 ± 0.04 ^de^	35.81 ± 0.07 ^bc^	110.95 ± 2.61 ^c^	81.01 ± 2.06 ^fg^	452.59 ± 39.72 ^b^

Same letters represent non-significant differences between treatments (LSD, α ≤ 0.05); HP = hydrolysable phenols; CP = condensed phenols; TPC = total phenolic compounds; gdm = gram of dry mass; mgGAE = milligram gallic acid equivalents; mgCE = milligram catechin equivalents; mgTE = milligrams trolox equivalents; mgFe^2+^ = milligram ferrous ion; results are the mean (n = 3) ± standard deviation.

**Table 6:** Effect of moisture, inoculum, and aeration on the phenolic content (HP, CP, and TPC) and the AC (DPPH, ABTS, and FRAP) of fermented PGH extracts.

Factor/ Measured Variables	Phenolic Content			Antioxidant Capacity		
	HP	CP	TPC	ABTS	DPPH	FRAP
Moisture (%)	+	+	+	+	+	+
Inoculum (sp/mL)	+	+ ^NS^	+	− ^NS^	+	− ^NS^
Aeration (L/Kgwm min)	−	−	−	− ^NS^	−	−

+ = positive effect; − = negative effect; α = 0.05; ^NS^ = Not significant; the results correspond to three replications.

**Table 7:** Phenolic compound profile of purified PGH extract.

Retention Time (min)	Mass [M-H]^−^ (M/Z)	Compound	Family
5.69	330.9	Gallic acid 4-*O*-glucoside	Hydroxybenzoic acids
19.31	950.5	Geraniin	Ellagitannins

## Data Availability

The original contributions presented in this study are included in the article. Further inquiries can be directed to the corresponding authors.
